# Deep learning to diagnose cardiac amyloidosis from cardiovascular magnetic resonance

**DOI:** 10.1186/s12968-020-00690-4

**Published:** 2020-12-07

**Authors:** Nicola Martini, Alberto Aimo, Andrea Barison, Daniele Della Latta, Giuseppe Vergaro, Giovanni Donato Aquaro, Andrea Ripoli, Michele Emdin, Dante Chiappino

**Affiliations:** 1 Deep Health Unit, Fondazione Toscana Gabriele Monasterio, Pisa-Massa, Italy; 2 Institute of Life Sciences, Scuola Superiore Sant’Anna, Pisa, Italy; 3 Cardiology Division, Fondazione Toscana Gabriele Monasterio, Pisa, Italy

**Keywords:** Deep learning, Artificial intelligence, Diagnosis, Amyloidosis, Cardiovascular magnetic resonance

## Abstract

**Background:**

Cardiovascular magnetic resonance (CMR) is part of the diagnostic work-up for cardiac amyloidosis (CA). Deep learning (DL) is an application of artificial intelligence that may allow to automatically analyze CMR findings and establish the likelihood of CA.

**Methods:**

1.5 T CMR was performed in 206 subjects with suspected CA (n = 100, 49% with unexplained left ventricular (LV) hypertrophy; n = 106, 51% with blood dyscrasia and suspected light-chain amyloidosis). Patients were randomly assigned to the training (n = 134, 65%), validation (n = 30, 15%), and testing subgroups (n = 42, 20%). Short axis, 2-chamber, 4-chamber late gadolinium enhancement (LGE) images were evaluated by 3 networks (DL algorithms). The tags “amyloidosis present” or “absent” were attributed when the average probability of CA from the 3 networks was ≥ 50% or < 50%, respectively. The DL strategy was compared to a machine learning (ML) algorithm considering all manually extracted features (LV volumes, mass and function, LGE pattern, early blood-pool darkening, pericardial and pleural effusion, etc.), to reproduce exam reading by an experienced operator.

**Results:**

The DL strategy displayed good diagnostic accuracy (88%), with an area under the curve (AUC) of 0.982. The precision (positive predictive value), recall score (sensitivity), and F1 score (a measure of test accuracy) were 83%, 95%, and 89% respectively. A ML algorithm considering all CMR features had a similar diagnostic yield to DL strategy (AUC 0.952 vs. 0.982; p = 0.39).

**Conclusions:**

A DL approach evaluating LGE acquisitions displayed a similar diagnostic performance for CA to a ML-based approach, which simulates CMR reading by experienced operators.

## Background

Amyloidosis is a systemic disorder characterized by the extracellular deposition of circulating proteins into amyloid fibers. These proteins are monoclonal light chains or transthyretin in the two most common forms, namely AL and ATTR amyloidosis, respectively [1]. Cardiac involvement is common in both AL and ATTR amyloidosis, predicts a worse outcome, and has important implications for treatment [1].

The diagnostic work-up for cardiac amyloidosis (CA) begins with the identification of clinical features, electrocardiographic (ECG) and imaging findings suggestive or compatible with CA, and often requires the histological demonstration of amyloid deposition, except when diphosphonate scintigraphy shows an intense myocardial uptake (Perugini scores 2-3) in the absence of a monoclonal gammopathy [2]. Despite its unique capability of allowing myocardial tissue characterization, the role of cardiovascular magnetic resonance (CMR) in this diagnostic flowchart is not well defined [2]. Several CMR findings are quite specific for CA, most notably a pattern of variable biventricular pseudohypertrophy with diffuse subendocardial-to-transmural late gadolinium enhancement (LGE). The degree of wall thickness increase and LGE extent are correlated to the degree of myocardial infiltration by amyloid fibers. Indeed, amyloid deposition is confined to the subendocardium of few myocardial segments in early disease stages and becomes more diffuse in patients with more advanced disease [3]. In the later stages, amyloid infiltration is so extensive that myocardial and blood-pool gadolinium kinetics are completely deranged, with diffuse gadolinium retention in the myocardium and an accelerated gadolinium washout from the bloodpool. This may cause problems in identifying the best inversion time (TI) of the myocardium in post-contrast images to obtain good LGE images. In these cases, TI-scout sequences [4], early-to-late enhancement acquisitions [5], phase sensitive inversion recovery (PSIR) LGE sequences [6], as well as native T1 mapping [7] and extracellular volume fraction (ECV) [8] may be helpful to establish the diagnosis and define the disease stage. Conversely, LGE areas might be very limited in the earlier disease stages, and lead to erroneous diagnoses of other ischemic or nonischemic cardiac disorders. We may add that several other cardiac and extracardiac findings (such as pericardial and pleural effusion) can be found in CA, but are not specific for this condition. Overall, the ability of human readers to diagnose CA is limited by the highly variable appearance of the disease across different stages, the technical difficulties related to the peculiar gadolinium kinetics in more advanced stages and is highly dependent on operator experience. These possible limitations of human reading might be overcome by using the tools of artificial intelligence (AI).

Machine learning (ML) algorithms build a mathematical model based on sample data, known as "training data", in order to make predictions or decisions without being explicitly programmed to perform the task. Automated ML analysis is faster with similar precision to the most precise human techniques [9]. Deep learning (DL) is a subset of ML using "raw data" to automatically identify salient features by means of a series of hierarchical representation levels that are not directly designed by humans, as in the case of ML. By avoiding the need for pre-processing techniques based on a priori knowledge of the human operator, DL allows the automatic extraction of salient information from "raw data" by using a series of levels of representation. In the field of medical imaging, integration of DL-based predictive analytics within clinical imaging is a natural order of progression wherein developments in cardiovascular imaging now provide high-fidelity datasets that possess more data than those acquired from prior generation scanners [10, 11]. The integration of DL-based algorithms with clinical imaging holds the promise to automate redundant tasks and improve disease diagnoses and prognostication, as well as to provide new insights into novel biomarkers associated with specific disease processes [12].

In the present study we tested the diagnostic performance of CMR-based ML and DL strategies in CA, focusing on conventional LGE images acquired using standardized parameters in a specialized CMR center.

## Methods

### Patient population

We evaluated 206 consecutive patients referred to an amyloidosis center (Fondazione Toscana Gabriele Monasterio, Pisa, Italy) between 2009 and 2019 because of suspected CA. All patients had signs and symptoms of cardiac disease, clinical and echocardiographic features deemed compatible with CA, and either a monoclonal gammopathy (n = 100, 49%), or an unexplained increase in left ventricular (LV) wall thickness on echocardiogram (interventricular septal thickness or inferolateral wall thickness at end-diastole ≥ 12 mm) (n = 106, 51%) [13]. Exclusion criteria were the presence of CMR-unsafe devices, and an estimated glomerular filtration rate < 30 mL/min/1.73 m^2^.

Patients underwent a complete diagnostic work-up including clinical evaluation, 12-lead ECG, transthoracic echocardiogram, CMR, serum and urine biochemistry comprising N-terminal pro-B-type natriuretic peptide, high sensitivity troponin T, serum free light chain assay along with serum and urine immunofixation-electrophoresis and myocardial or non-myocardial biopsy [2]. Patients with suspected ATTR cardiomyopathy also underwent diphosphonate scintigraphy. The study protocol conformed to the 1975 Declaration of Helsinki [14], and was approved by the Institutional Human Research Committee. All patients provided written informed consent.

### Diagnosis of cardiac amyloidosis

CA was diagnosed in 107 subjects (52% of the whole population; 50 AL, 57 ATTR amyloidosis). Cardiac AL amyloidosis was defined by an endomyocardial biopsy (EMB) containing AL amyloid (n = 35, 70%), or the combination of characteristic features on echocardiography/CMR and histologically proven systemic AL amyloidosis on a non-cardiac biopsy (n = 15, 30%)[15]. Cardiac ATTR amyloidosis was diagnosed when patients had an EMB containing ATTR amyloid (n = 30, 53%), or grade 2–3 cardiac uptake on diphosphonate scintigraphy in the absence of monoclonal gammopathy (n = 27, 47%) [2]. The large majority of patients (n = 55, 96%) had wild-type ATTR.

### Cardiovascular magnetic resonance

Patients underwent 1.5 T CMR examination (Signa Excite, GE Healthcare, Waukesha, Wisconsin, USA, n = 156, 76%; Signa Artist, GE Healthcare, n = 50, 24%) using an 8-channel and a 32-channel phased-array surface receiver coils respectively and vectorcardiogram triggering. Biventricular systolic function was assessed by breath-hold balanced steady-state free precession cine imaging in the short-axis (SAx) stack (8-mm thickness, no gap). Sequence parameters were: field-of-view 380–400 mm, repetition/echo time 3.2/1.6 ms, flip angle 60°, matrix 224 × 192, phases 30. LGE imaging was performed between 10 and 20 min after contrast agent administration (Gadoteric acid, DOTAREM, 0.2 mmol/kg; Guerbet, Villepinte, France) using a segmented T1-weighted gradient-echo inversion-recovery pulse sequence. In SAx orientation, the LV was completely encompassed by contiguous 8-mm thick slices (with no inter-slice gap). LGE was also confirmed or excluded in vertical and horizontal long-axis views. TI was individually adapted to suppress the signal of normal remote myocardium (220–300 ms); sequence parameters were: field-of-view 380–400 mm, slice thickness 8 mm, repetition/echo time 4.6/1.3 ms, flip angle 20º, matrix 256 × 192. In all cases, a midventricular short-axis TI-scout sequence was used to choose the appropriate inversion time and to check the presence of paradoxical blood/myocardium TI; moreover, according to an algorithm previously validated by our group [5], LGE images were preceded by 5–10 early enhancement images acquired every minute after contrast injection with a fixed TI (typically 250 ms, as for LGE images) until the normal myocardium nulls, in order to track Gd wash-in and wash-out kinetics into the bloodpool and into the myocardium, respectively. Both procedures allow the radiographer to choose the right TI (the one that makes the diseased myocardium bright) for LGE acquisition, even in cases of extensive CA and paradoxical TI.

All CMR studies were analyzed off-line using a workstation (Advantage Workstation, GE Healthcare) with a dedicated software (MASS 6.1, Medis, Leiden, Netherlands) by an experienced CMR reader (A.B.). Using the stack of SAx cine images, left ventricular (LV) and right ventricular (RV) volumes, mass and global function were calculated [16]. In all 17 segments (according the American Heart Association/American College of Cardiology classification) [17], the presence and pattern of LGE (subendocardial, midwall, subepicardial, transmural) were assessed visually; patients with only faint LGE areas limited to the RV insertion points were considered as LGE-negative. A diffuse subendocardial-to-transmural pattern (i.e. LGE with a subendocardial or transmural pattern, involving more than 5 contiguous LV segments, not related to a definite coronary artery distribution) [13] was considered suggestive of CA. Blood-pool early darkening was defined as an abnormal darkness of the blood-pool in LGE images, i.e. a signal intensity decay > 50% during the first 10 min after Gd injection at LGE images acquired with a fixed TI, because of accelerated Gd wash-out from the blood-pool in patients with diffuse extracellular amyloid deposition [5].

### Descriptive statistics

Descriptive statistics were performed using SPSS Statistics (version 22, Statistical Package for the Social Sciences, International Business Machines, Inc., Armonk, New York, USA). Normal distribution was assessed through the Kolmogorov–Smirnov test; as all variables had non-normal distribution, they were expressed as median and interquartile intervals. Differences between groups were tested through the Mann–Whitney U test. Categorical variables were compared by the Chi-square test with Yates correction. p values < 0.05 were considered statistically significant.

### Deep learning approach

#### Network architecture

LGE images in different orientations were input into 3 base convolutional neural networks (CNNs: 2C, 4C, SAx). The global CNN established the likelihood of CA based on the average prediction scores from the 3 individual CNNs. Each CMR examination was classified as belonging to the “amyloidosis” subset when the estimated probability was ≥ 0.5, or to the “no amyloidosis” subset when the probability was < 0.5.

The architecture of the base CNN is depicted in Additional file 1: Figure S1. The CNN included a total of 4 convolutional layers of 64, 128, 256, 512 filters with kernel size of 3 × 3 and stride of 2. Each convolutional layer was followed by a batch normalization layer and then by an activation layer with the Rectified Linear Unit (ReLU). The 512 feature maps of the last convolutional layer were averaged using a Global Average Pooling layer and were followed by a dropout layer with dropout rate of 0.2. A fully connected layer with 16 units provided the high-level features for each LGE orientation. These features were input to the last fully connected layer with a final softmax layer that provided the predicted probability associated to the binary classification. The base CNN was pretrained on a large CMR dataset (> 90,000 series) with a self-supervised dual task for the identification of the image type (cine, LGE and black-blood) and orientation (2- and 4-chambers [2C, 4C], SAx). A transfer learning approach was then applied to fine-tune the CNN on the CA dataset.

#### Network training

Patients were randomly assigned to 3 subgroups, which were used for training (n = 134, 65%), internal validation (n = 30, 15%), and testing (n = 42, 20%) of the global CNN. The validation step was devised to tune the network hyperparameters and select the model. Image augmentation was performed online on training data by applying random translations, rotations and flipping and by adding random noise. Different learning strategies (data augmentation, batch normalization in convolutional layers, dropout before dense layers) were adopted to prevent model overfitting. In addition, we used a relatively small batch size (m = 24) combined to a low learning rate of 1*10^–5^ to improve training stability and generalization performance. Binary cross entropy was used as loss function. After training for 5000 epochs, the model with the best performance on the validation dataset was chosen. All neural network implementation and training was performed with high-level DL library Keras of Tensorflow v2.0.

#### Activation maps

To allow an easier interpretation of image feature extraction, we derived activation maps using the Gradient-weighted Class Activation Mapping (Grad-CAM) technique [18]. These heatmaps highlight the portion of the input image that contributed most for the category predictions. Grad-CAM maps were obtained projecting back the weights of the output layer on the convolutional feature maps obtained from the last convolution layer (layer Conv4 in Additional file 1: Figure S1).

### Machine learning of human-extracted variables

A gradient boosting machine (GBM) model was built for the binary classification of patients (CA vs no CA) based on clinical and imaging features extracted from the CMR exam. We performed an exhaustive search over GBM model parameters using five-fold cross validation. The maximum number of trees was set to 40, the maximum tree depth was set to 2, and the maximum learning rate to 0.1 and the minimum samples of leaf to 2. The best model was tested on the same test dataset used for the deep learning approach. Performance was assessed by the area under the curve (AUC) of the receiver operating characteristic. Permutation-based feature importance was computed to examine which variable had the most predictive power. GBM model implementation and analysis were performed in Python 3.6.9 with the machine learning library scikit-learn v0.22.

## Results

### Population characteristics

At the end of the diagnostic work-up, CA was diagnosed in 107 subjects (52% of the whole population; 50 AL, 57 ATTR amyloidosis). The final diagnoses in the other patients (n = 99, 48%) were hypertensive cardiomyopathy (n = 39, 39%), valve heart disease (n = 20, 20%), hypertrophic cardiomyopathy (n = 8, 7%), blood dyscrasia or AL amyloidosis without cardiac involvement (n = 28, 28%), or unclear or multiple mechanisms (n = 4, 4%). A history of previous myocardial infarction (MI) was present in 6 patients with CA and in 13 patients without CA. Patients with CA had higher LV mass index (LVMI), had more frequently LGE, had circumferential subendocardial or diffuse LGE, early blood-pool darkening and pleural effusion than patients without CA (Table 1). The clinical data and CMR findings in the training, validation and testing subgroups were homogeneous (Table 2).

**Table 1:** Patient characteristics in the subgroups with or without cardiac amyloidosis (CA)

	All n = 206	CA n = 107 (52%)	No CA n = 99 (48%)	*p-value
Age (years)	76 (69–81)	76 (69–81)	73 (65–79)	0.178
Male sex (n, %)	137 (67)	79 (74)	58 (59)	*0.021*
BSA (m^2^)	1.84 (1.74–1.96)	1.84 (1.72–1.94)	1.85 (1.75–2.01)	0.399
CMR findings				
LVEDVI (mL/m^2^)	79 (67–96)	79 (67–92)	81 (67–97)	0.153
LVESVI (mL/m^2^)	33 (23–50)	31 (24–46)	36 (22–56)	0.445
LVSVI (mL/m^2^)	44 (37–51)	43 (36–51)	45 (39–51)	0.192
LVEF (%)	55 (46–67)	57 (46–65)	55 (44–67)	0.625
CO (L/min)	5.3 (4.3–6.6)	5.3 (4.3–6.5)	5.2 (4.4–6.7)	0.752
LVMI (g/m^2^)	84 (71–111)	95 (77–118)	73 (64–98)	* < 0.001*
LA area index (cm^2^/m^2^)	16 (13–18)	17 (14–18)	14 (12–18)	*0.008*
RA area index (cm^2^/m^2^)	13 (11–15)	14 (12–16)	12 (10–14)	* < 0.001*
RVEDVI (mL/m^2^)	70 (57–81)	74 (60–83)	63 (55–77)	*0.013*
RVESVI (mL/m^2^)	28 (20–37)	32 (22–41)	24 (18–32)	* < 0.001*
RVSVI (mL/m^2^)	39 (33–47)	38 (32–43)	41 (36–49)	0.087
RVEF (%)	59 (47–65)	54 (44–63)	61 (57–68)	* < 0.001*
Early bloodpool darkening (n, %)	40 (19)	39 (36)	1 (1)	* < 0.001*
LGE presence (n, %)	174 (84)	105 (98)	69 (69)	* < 0.001*
LGE subendocardial-to-transmural pattern (n, %)	89 (43)	85 (79)	4 (4)	* < 0.001*
Pericardial effusion (n, %)	48 (23)	30 (28)	18 (18)	0.087
Pleural effusion (n, %)	58 (28)	45 (42)	13 (13)	* < 0.001*

*AL* amyloid light-chain, *ATTR* amyloid transthyretin, *BSA* body surface area, *CMR* cardiovascular magnetic resonance, *CO* cardiac output, *LA* left atrium, *LGE* late gadolinium enhancement, *LVEDVI* left ventricular end-diastolic volume index, *LVESVI* left ventricular end-systolic volume index, *LVEF* left ventricular ejection fraction, *LVMI* left ventricular mass index, *LVSVi* left ventricular stroke volume index, *RA* right atrium, *RVEDVI* right ventricular end-diastolic volume index, *RVESVI* right ventricular end-systolic volume index, *RVEF* right ventricular ejection fraction, *RVSVI* right ventricular stroke volume index

*p-values represent the difference between patients with CA and without CA (no CA), p-values less than 0.05 are shown in italic

**Table 2:** Population characteristics: training, validation and test subgroups

	All n = 206	Training subgroup n = 134 (65%)	Validation subgroup n = 30 (15%)	Test subgroup n = 42 (20%)	p-value
Age (years)	76 (69–81)	77 (69–81)	71 (65–79)	73 (63–79)	0.097
Male sex (n, %)	137 (67)	88 (66)	20 (67)	29 (69)	0.623
BSA (m^2^)	1.84 (1.74–1.96)	1.83 (1.73–1.95)	1.88 (1.75–2.02)	1.85 (1.76–1.95)	0.206
Amyloidosis (n, %)	107 (52)	71 (53)	15 (50)	21 (50)	0.645
AL/ATTR/no amyloidosis (n, %)	50/57/99 (24/28/48)	33/38/63 (25/28/47)	6/9/15 (20/30/50)	11/10/21 (26/24/50)	0.610
CMR findings					
LVEDVI (mL/m^2^)	79 (67–96)	79 (64–95)	71 (66–92)	84 (70–106)	0.105
LVESVI (mL/m^2^)	33 (23–50)	31 (23–50)	27 (20–42)	40 (26–56)	0.112
LVSVI (mL/m^2^)	44 (37–51)	42 (37–49)	44 (39–52)	47 (38–54)	0.131
LVEF (%)	55 (46–67)	56 (47–67)	62 (51–70)	53 (45–64)	0.277
CO (L/min)	5.3 (4.3–6.6)	5.1 (4.3–6.3)	5.5 (5.1–7.3)	5.5 (4.4–7.0)	0.565
LVMI (g/m^2^)	84 (71–111)	88 (71–114)	82 (70–113)	82 (73–107)	0.730
LA area index (cm^2^/m^2^)	16 (13–18)	15 (13–18)	17 (13–18)	16 (14–18)	0.714
RA area index (cm^2^/m^2^)	13 (11–15)	13 (11–15)	13 (10–17)	13 (12–15)	0.742
RVEDVI (mL/m^2^)	70 (57–81)	66 (55–80)	71 (55–89)	73 (62–81)	0.212
RVESVI (mL/m^2^)	28 (20–37)	28 (20–37)	27 (19–34)	30 (22–37)	0.180
RVSVI (mL/m^2^)	39 (33–47)	38 (33–44)	40 (34–52)	42 (34–51)	0.624
RVEF (%)	59 (47–65)	59 (49–64)	60 (49–67)	60 (47–65)	0.468
Early darkening (n, %)	40 (19)	26 (19)	8 (27)	6 (14)	0.241
LGE presence (n, %)	174 (84)	115 (86)	25 (83)	34 (81)	0.954
LGE subendocardial-to-transmural pattern (n, %)	89 (43)	61 (46)	11 (37)	18 (43)	0.434
Pericardial effusion (n, %)	48 (23)	32 (22)	4 (13)	12 (29)	0.465
Pleural effusion (n, %)	58 (28)	39 (27)	7 (23)	12 (29)	0.606

*AL* amyloid light-chain, *ATTR* amyloid transthyretin, *BSA* body surface area, *CMR* cardiovascular magnetic resonance, CO cardiac output, *LA* left atrium, *LGE* late gadolinium enhancement, *LVEDVI* left ventricular end-diastolic volume index, *LVESVI* left ventricular end-systolic volume index, *LVEF* left ventricular ejection fraction, *LVMI* left ventricular mass index, *LVSVI* left ventricular stroke volume index, *RA* right atrium, *RVEDVI* right ventricular end-diastolic volume index, *RVESVI* right ventricular end-systolic volume index, *RVEF* right ventricular ejection fraction, *RVSVI* right ventricular stroke volume index

### Deep learning approach to diagnose cardiac amyloidosis

**Fig. 1:**
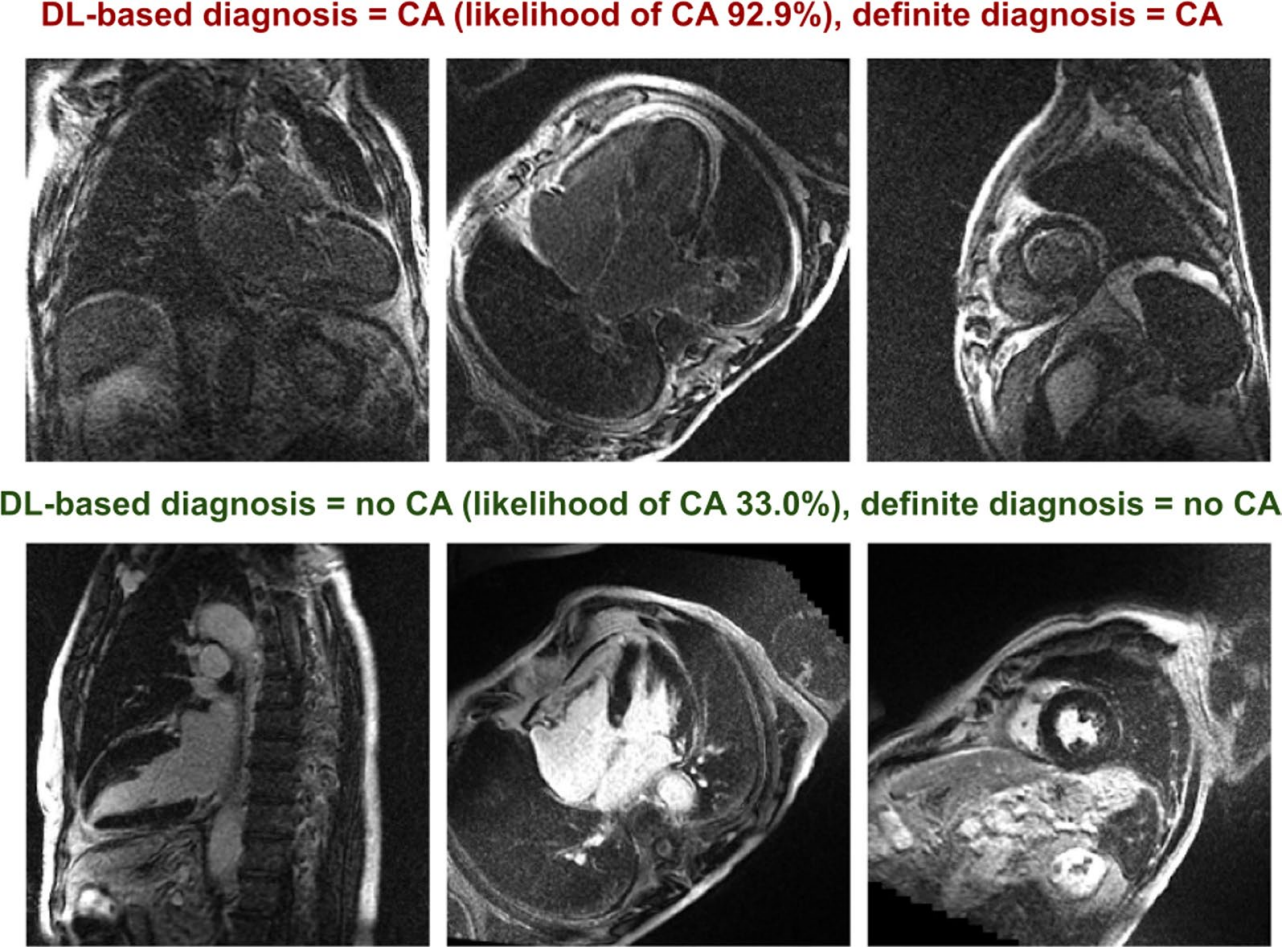
Two examples of adjudication of the diagnosis of cardiac amyloidosis (CA) using the deep learning approach. These 2 patients (a 75-year old man, above, and a 68-year-old man, below) were correctly acknowledged as having CA or not being affected by this disorder, respectively

**Fig. 2:**
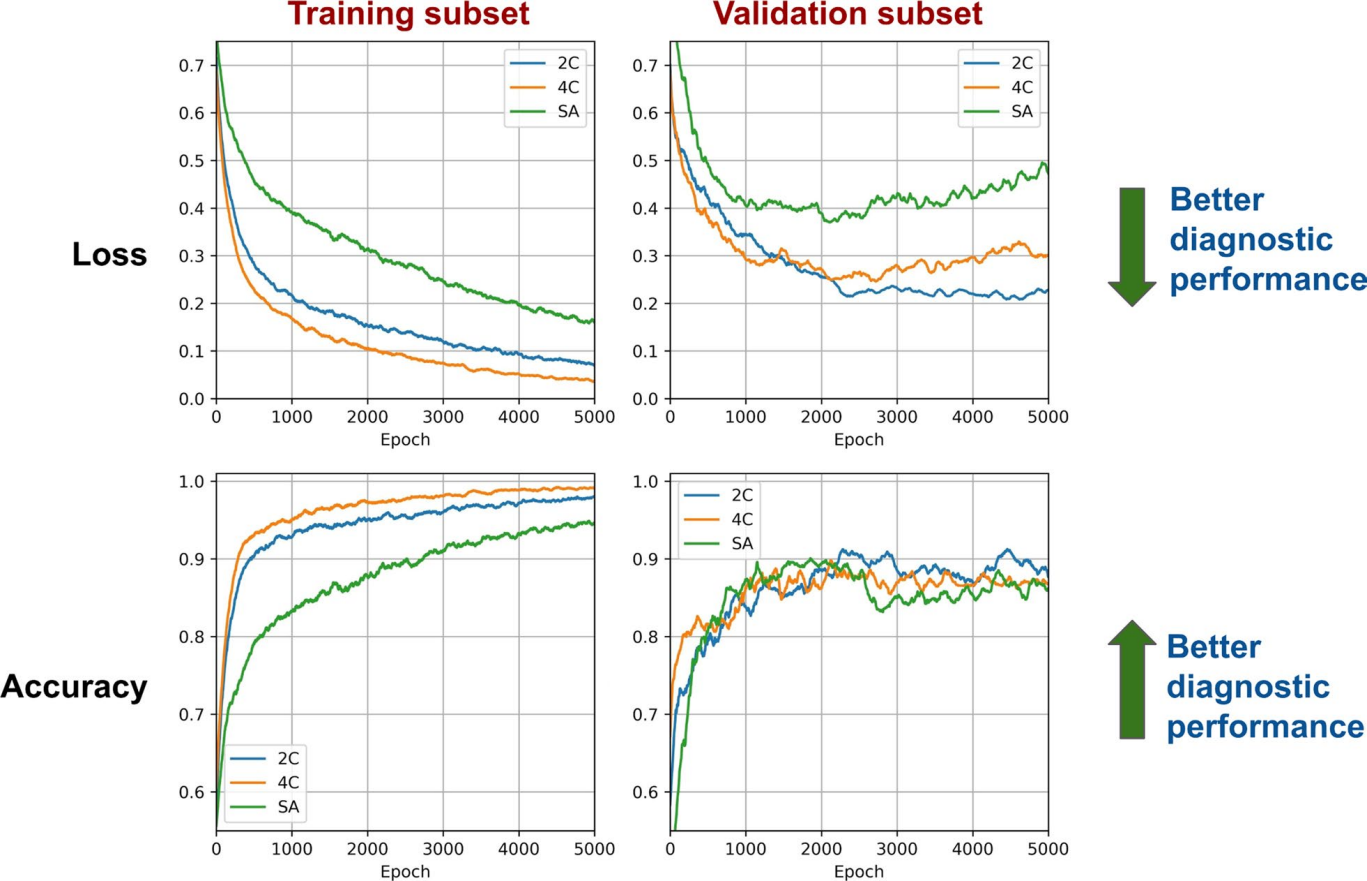
Loss and accuracy curves of the convolutional neural network in the training and validation subsets. The trends of the curves denoted a good diagnostic performance of the deep learning method, with no evidence of overfitting

**Fig. 3:**
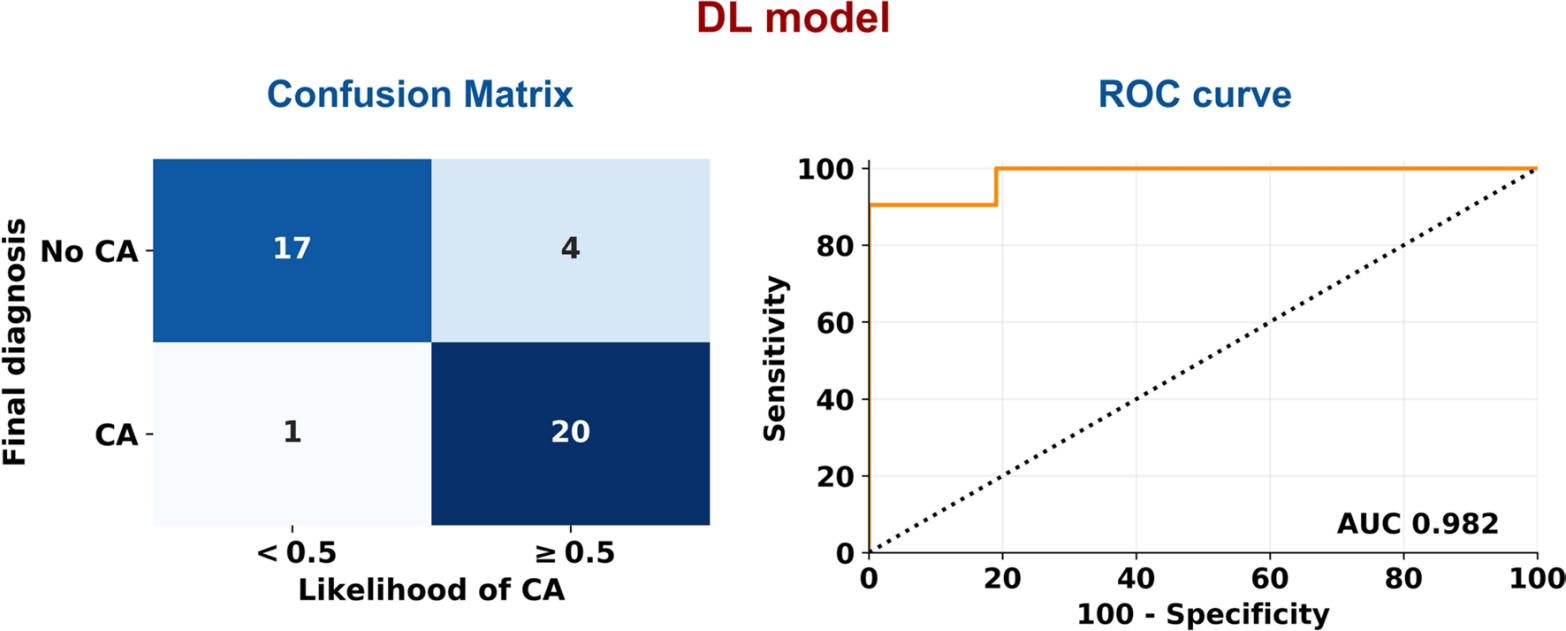
Diagnostic performance of the convolutional neural network. *AUC* area under the curve, *CA* cardiac amyloidosis, *DL* deep learning, *ROC* receiver operating characteristics

**Fig. 4:**
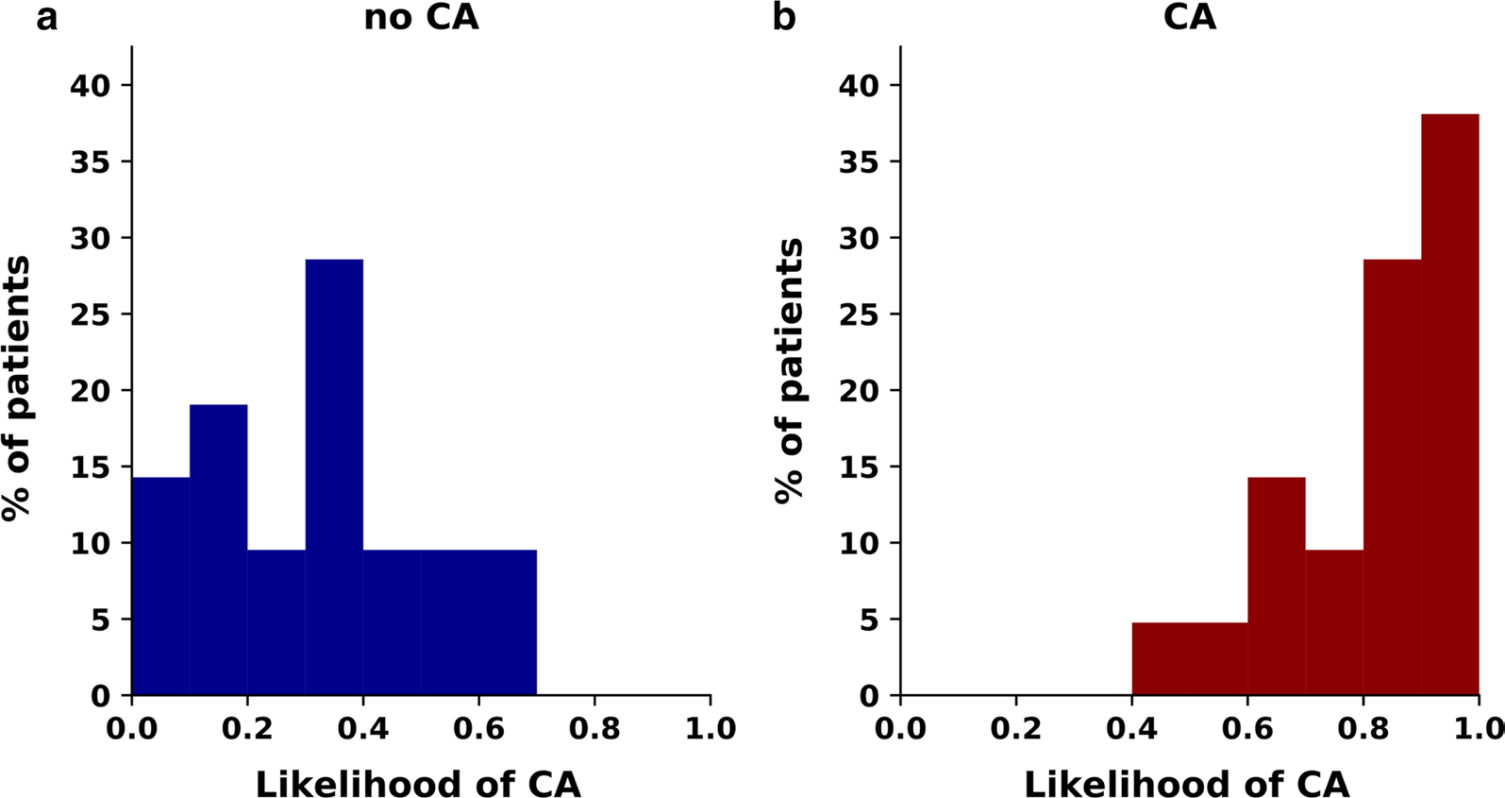
Patient stratification by deciles of likelihood of cardiac amyloidosis (CA), according to the final diagnosis (no CA vs. CA)

The likelihood of CA was defined based on a CNN considering 2C, 4C and SAx LGE images (Fig. 1). Loss and accuracy curves in the training and validation subsets are shown in Fig. 2. In the testing subgroup, the CNN had an 88% accuracy, as it correctly classified 37 of 42 patients , with 4 false positive and 1 false negative results (Fig. 3). Sixty-six percent of patients had a likelihood of 0.8–1.0, and none of the patients ultimately diagnosed with CA had a likelihood of disease lower than 0.4 (Fig. 4). Notably, a likelihood of 0.4 could be used also to exclude CA, having a negative predictive value of 100% (Table 3). Other measures of diagnostic performance are reported in Table 4. Notably, the CNN had an AUC of 0.982 for the diagnosis of CA. The 4 myocardial infarction patients from the dataset test were all correctly classified.

**Table 3:** Patient stratification by deciles of likelihood of cardiac amyloidosis (CA), according to the final diagnosis (no CA vs. CA)

Likelihood of CA	False Positive (n)	False Negative (n)	Sensitivity (%)	Specificity (%)	PPV (%)	NPV (%)
0.1	18	0	100	14	54	100
0.2	14	0	100	33	60	100
0.3	12	0	100	43	64	100
0.4	6	0	100	71	78	100
0.5	4	1	95	81	83	94
0.6	2	2	90	90	90	90
0.7	0	5	76	100	100	81
0.8	0	7	67	100	100	75
0.9	0	13	38	100	100	62

*NPV* negative predictive value, *PPV* positive predictive value

**Table 4:** Deep learning (DL) vs. machine learning (ML)-based algorithms for the diagnosis of cardiac amyloidosis

	DL method	ML method
Accuracy (%)	88%	90%
Precision score (%)	83%	95%
Recall score (%)	95%	86%
F1 score (%)	89%	90%
AUC	0.982	0.952

*AUC* area under the curve

**Fig. 5:**
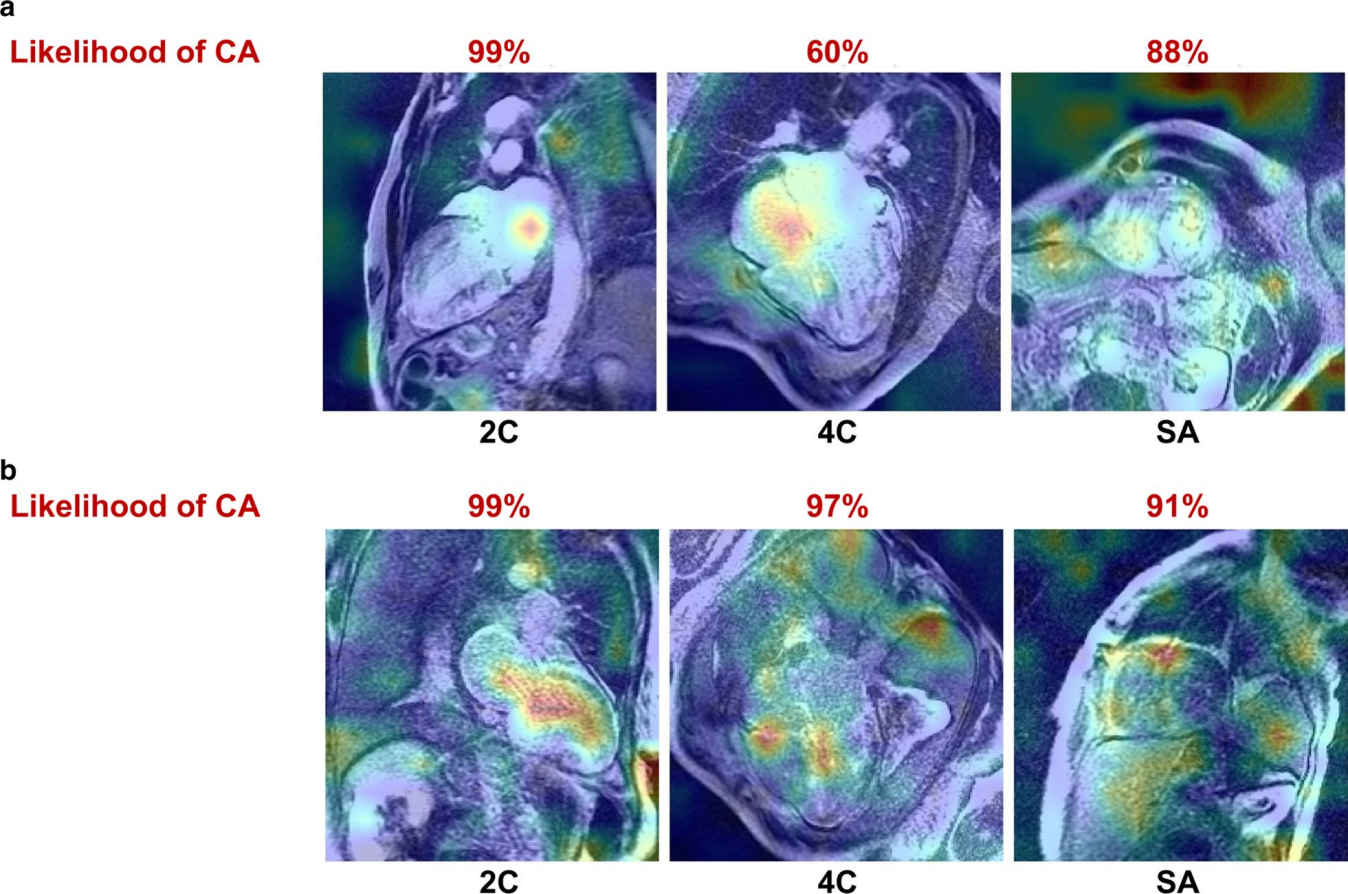
Activation maps from two patients showing the most informative image elements in two cardiovascular magnetic resonance examinations. *2C* two-chamber, *4C* four-chamber, *CA* cardiac amyloidosis, *SA* short axis

Activation maps produced by the Grad-CAM analysis showed that the most informative image features for CA prediction were located within the heart or in elements related to CA, such as pleural effusion (Fig. 5).

### Deep learning versus simulation of human reading

**Fig. 6:**
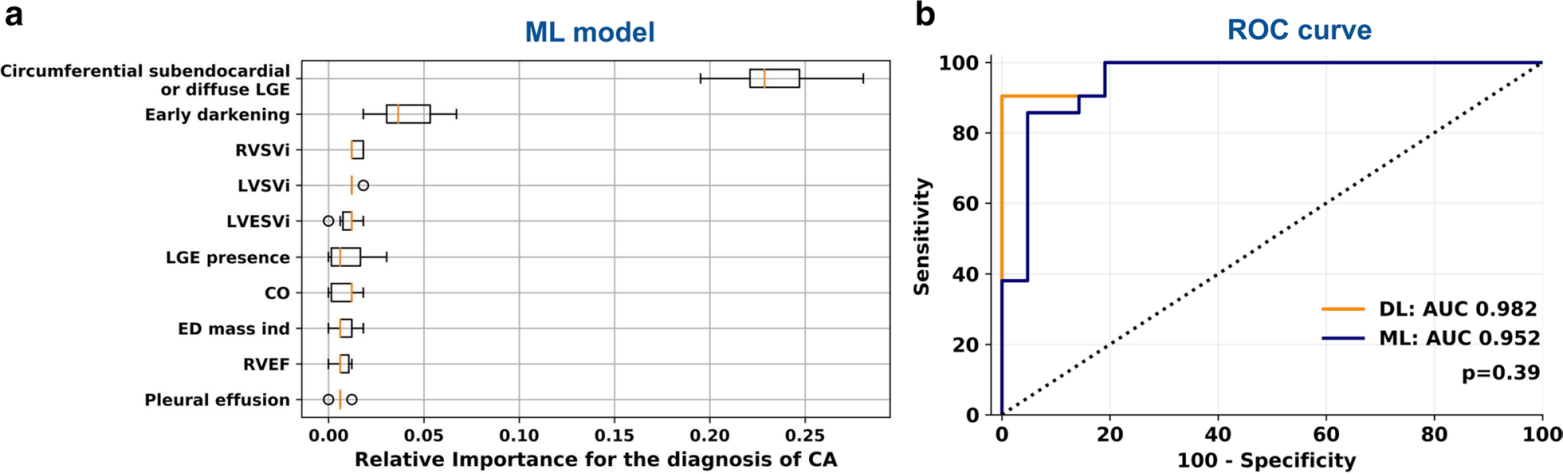
Manually extracted features included in the machine learning (ML)-based algorithm (**a**), and comparison of the ROC curve with the deep-learning (DL)-based approach (**b**). *AUC* area under the curve, *BSA* body surface area, *CA* cardiac amyloidosis, *CO* cardiac output, *LA* left atrium, *LGE* late gadolinium enhancement, *LVEDVI* left ventricular end-diastolic volume index, *LVEF* left ventricular ejection fraction, *LVESVI* left ventricular end-systolic volume index, *LVMI* left ventricular mass index, *LVSVI* left ventricular stroke volume index, *RA* right atrium, *ROC* receiver operating characteristics, *RVEDVI* right ventricular end-diastolic volume index, *RVESVI* right ventricular end-systolic volume index, *RVSVI* right ventricular stroke volume index

All CMR features deriving from manual extraction (biventricular volumes and function, LGE presence and pattern, early darkening, pericardial and pleural effusion) were then considered in a ML model based on the gradient boosting classifier. This model correctly classified 38 of 42 patients, with an accuracy of 90%, and an AUC of 0.952 (Table 4 and Fig. 6). Variable importance analysis showed that the circumferential subendocardial LGE pattern (considered as a binary variable: present vs. absent) was the strongest predictor of CA, followed by early blood-pool darkening (Fig. 6a). Based on a comparison between the AUC curves, the CNN and the ML-based method displayed a similar diagnostic performance (p = 0.39; Fig. 6b).

The inference time on CPU (Intel® Core i7-7800X CPU @ 3.50 GHz) for the DL analysis of the 42 patients of the test sample was 1.4 s, and the inference time on GPU (Nvidia GeForce® GTX 1080 1080 Ti—12 Gb) was 0.7 s. Conversely, a ML approach required input variables that were manually collected by an experienced operator; the time needed was difficult to quantify, but several orders of magnitude greater than the DL approach.

## Discussion

We report for the first time that an automated interpretation of CMR exams by a DL-based approach allows to reliably identify patients with CA with a high degree of accuracy. We evaluated a population of patients referred to CMR because of clinically suspected CA; among these patients, 52% were adjudicated as having CA by the standard diagnostic algorithm. A CNN establishing the likelihood of CA based on 2C, 4C and SAx LGE acquisitions was developed, and tested in a population subgroup, where it displayed an AUC of 0. 982. This approach had a similar diagnostic performance than a combination of manually extracted CMR features (p = 0.39 for the comparison of the AUC values) by a ML-based approach, which recapitulates CMR reading by experienced operators.

In the near future, AI applications are expected to “transform cardiac imaging” by “covering a range of applications from image classification, image reconstruction, automation in segmentation and quantification and guiding diagnosis and prognosis” [19]. In the diagnostic setting, AI can allow accurate image segmentation and automated measurements, can define the likelihood of a specific condition (such as obstructive coronary artery disease based on perfusion single-photon emission computed tomography [20, 21]), and may assist human readers in diagnosing cardiac disorders, from heart failure [22] to rare disorders such as CA, which may be misdiagnosed outside of referral centers. An AI-based diagnostic tool would quantify the likelihood of CA based on automated image analysis, and could simplify human interpretation of CMR examinations. To create such a tool, we trained a neural network to establish the likelihood of CA based on the most relevant features from LGE images, which we considered the most relevant acquisitions for diagnostic purposes; this assumption was confirmed by a dedicated analysis showing that a circumferential subendocardial or diffuse LGE pattern is the strongest predictor of CA, followed by LGE presence (Fig. 6a). In the testing subset, the CNN displayed a good diagnostic performance, with an AUC value approaching 1, and a satisfactory accuracy (88%). Importantly, among the 5 patients incorrectly classified by the CNN, there was only 1 false negative: should we have decided whether or not to perform further diagnostic investigations based on automated CMR interpretation, we would have missed only one CA case of 42 patients evaluated (2%). A lower threshold for the likelihood of CA (0.4) had 100% negative predictive value, thus being an ideal threshold to exclude CA.

The fact that only LGE sequences were evaluated through the CNN may be questioned. Nonetheless, it is important to consider that all imaging data from LGE acquisitions were considered, and not LGE patterns alone. While a CNN can be assimilated to a black box, some hints of its functioning are provided by attention maps, which suggested the evaluation of myocardial walls, as well as of the blood-pool, and even of extracardiac findings such as pericardial and pleural effusion (Fig. 5b). According to the training curves (Fig. 2), the LGE acquisition in four-chamber view seemed to be the most informative, possibly because it explores not only the whole heart (including both atria and the right ventricle), but also the presence of pericardial and pleural effusions. We also compared the diagnostic performance of our CNN with image analysis by experienced CMR readers, which was simulated by including manually delineated LV, RV and atrial contours (from which several parameters associated with chamber volumes, mass and function could be calculated), and several categorical variables (LGE presence and pattern, presence of early blood-pool darkening, etc.; Fig. 6a). When assessing the likelihood of CA based on the combination of these findings, which recapitulates the process of CMR interpretation by human readers, this process displayed a similar diagnostic yield than the CNN, with no significant differences between AUC values at discrimination analysis.

These findings corroborate the conclusion that the DL algorithm (which could be easily implemented as a software for automated image analysis) may provide a valuable support to CMR reading when patients are referred for suspected CA. The two main advantages of DL are speed and accuracy. Accuracy appears similar to ML (and likely an experienced observer), but the speed advantage is unquestionable.

In our analysis, we included 19 patients with prior myocardial infarction, all presenting typical regional wall motion abnormalities and subendocardial-to-transmural LGE in the infarcted areas. The DL and ML approaches were not affected by the presence of an ischemic scar, and all 4 patients with prior infarction in the test dataset were correctly classified.

Several limitations of this hypothesis-generating study must be acknowledged. First, sample size was small, although the loss and accuracy curves still displayed a good diagnostic accuracy with no evidence of overfitting (Fig. 2). Second, the prevalence of CA was very high (107 out of 206), and this diagnostic algorithm should be validated in non-specialized centers with a lower prevalence of CA. Third, the study would have also benefited from an external validation cohort with a good representation of patients with hypertensive heart disease, hypertrophic cardiomyopathy, cardiac sarcoidosis and other pathologies that could be mistaken for CA. Fourth, ML on imaging features was considered as a surrogate of expert reading blinded to the clinical data given the retrospective study design. Fifth, PSIR and parametric mapping (native T1 mapping and ECV quantification) were not implemented, because these techniques were not available for the earlier exams, and are still not available at all CMR centers. Sixth, the analysis focused only on CMR findings, and particularly on LGE images, while human interpretation of CMR examinations takes into also clinical data, ECG and echocardiographic findings, etc. On the other hand, the CNN could be easily implemented to consider additional variables for the purpose of diagnosing CA. Seventh, we considered AL and ATTR cardiomyopathies as a single diagnostic entity (CA), given the relatively small patient number. Eighth, the functioning of our DL-based system for image interpretation cannot be explained, by its very nature, unless partially (and in a patient-based fashion) by attention maps, which show which elements of the image are particularly important. Finally, our diagnostic system implies LGE acquisitions obtained at a single high-volume CMR lab using a conventional gradient-echo inversion-recovery sequence and carefully setting the TI to null the normal myocardium and to highlight (as bright) the affected myocardium. This approach is based on the acquisition of 5–10 early enhancement images every minute after contrast injection with a fixed TI as previously validated by our group [5], and on the acquisition of TI-scout sequences to choose the appropriate inversion time of the normal myocardium and to check the presence of paradoxical blood/myocardium TI; further studies are needed to test this algorithm across different LGE sequences (including PSIR LGE), different contrast doses and different TIs, causing a highly variable signal intensity in cases of CA if acquisition parameters are not standardized. Non-contrast CMR is attracting attention as a potential novel perspective for the diagnosis of CA [7, 23, 24], and should be considered in future studies.

## Conclusions

We report that a DL approach evaluating 2C, 4C and SAx LGE acquisitions displayed a similar diagnostic performance for CA than a ML-based approach, which simulated CMR reading by experienced operators. Further studies are needed to validate this algorithm in external centers, using larger populations and different LGE sequences.

## Supplementary information

**Additional file 1: Figure S1. **Convolutional Neural Network (CNN) architecture.

## Data Availability

The datasets used and/or analyzed during the current study are available from the corresponding author on reasonable request.

## Abbreviations

2C: 2-Chamber
4C: 4-Chamber
AI: Artificial intelligence
AL: Light-chain amyloidosis
AUC: Area under the curve
ATTR: Amyloid transthyretin
CA: Cardiac amyloidosis
CMR: Cardiovascular magnetic resonance
CNN: Convolutional neural network
DL: Deep learning
ECG: Electrocardiogram
EMB: Endomyocardial biopsy
GBM: Gradient boosting machine
Grad-CAM: Gradient-weighted class activation mapping
LGE: Late gadolinium enhancement
LV: Left ventricle/left ventricular
LVMI: Left ventricular mass index
ML: Machine learning
PSIR: Phase sensitive inversion recovery
ReLU: Rectified linear unit
RV: Right ventricle/right ventricular
SAx: Short axis
TI: Inversion time
