# Supplementary material for: Deep learning to diagnose cardiac amyloidosis from cardiovascular magnetic resonance
Source: J Cardiovasc Magn Reson. 2020 Dec 7;22:84. doi: 10.1186/s12968-020-00690-4 (PMC7720569; doi:10.1186/s12968-020-00690-4)
Supplement: Supplementary file 1 — Additional file 1: Figure S1. Convolutional Neural Network (CNN) architecture. [file 12968_2020_690_MOESM1_ESM.docx]

**Additional material**

**Additional Figure S1. Convolutional Neural Network (CNN) architecture.**


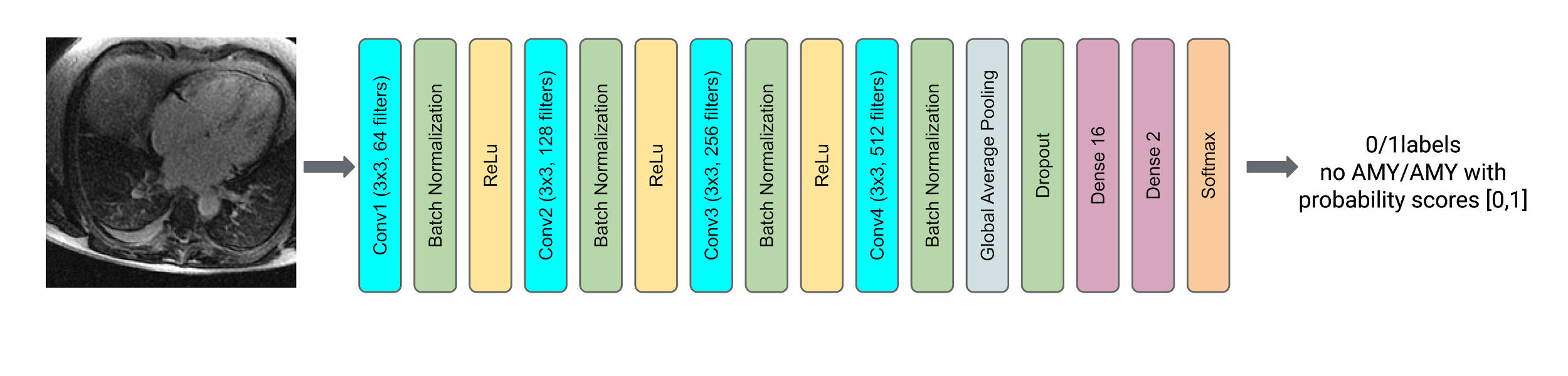


For details, see the **Methods** section. Conv, 2D convolutional layer with kernel 3x3 and stride 2, ReLU, Rectified Linear Unit layer, Dense, fully-connected layer.
